# Interventions for digital eye strain in adolescents: a structured review with supporting evidence from young adult populations

**DOI:** 10.3389/fpubh.2026.1942125

**Published:** 2026-09-10

**Authors:** Ioanna Mylona, Georgios D. Floros

**Affiliations:** 1 School of Medicine, Aristotle University of Thessaloniki, Thessaloniki, Greece; 2 Department of Ophthalmology, General Hospital of Serres, Serres, Greece; 3 2nd Department of Psychiatry, Aristotle University of Thessaloniki, Thessaloniki, Greece

**Keywords:** adolescents, computer vision syndrome, digital eye strain, ergonomic interventions, ocular surface, screen use, visual fatigue, young adults

## Abstract

**Objective:**

To review and synthesize interventions for digital eye strain in adolescents, while incorporating evidence from young adult populations to address gaps in the literature.

**Background:**

Digital eye strain (DES) is increasingly prevalent among adolescents due to the growing use of digital devices. However, evidence regarding effective interventions specifically targeting this population remains limited.

**Methods:**

A structured review of studies evaluating interventions for DES was conducted. Adolescents were defined as individuals under 18 years of age. Due to the paucity of adolescent-only studies, studies involving young adults (≥18 years) were also included where relevant. Interventions were categorized into optical, behavioral/ergonomic, pharmacological, nutritional, and educational approaches. Data on study design, population characteristics, and outcomes were extracted and summarized.

**Results:**

Few studies exclusively investigated adolescents, with most evidence derived from young adult populations or mixed-age cohorts. Interventions demonstrating benefit included behavioral and ergonomic strategies (e.g., optimized viewing distance, break schedules), ocular surface management (e.g., artificial tears), and selected optical or nutritional approaches. Blue-light filtering interventions generally showed limited or no benefit. Adolescent-specific studies, although scarce, indicated that both behavioral and nutritional interventions may reduce DES symptoms.

**Conclusion:**

There is a clear paucity of intervention studies targeting adolescents with digital eye strain. Evidence from young adult populations suggests that ergonomic and ocular surface-focused strategies may have relevance for adolescents; however, direct evidence in adolescent populations remains limited and age-specific studies are needed before definitive recommendations can be made.

## 1 Introduction

### 1.1 Rationale/background

Digital eye strain (DES), also known as computer vision syndrome (CVS), encompasses ocular and visual symptoms (e.g., eye fatigue, dry eyes, blurred vision, headaches, irritation) associated with prolonged digital screen use (1). Prevalence is high globally (approximately 66–69% across populations), with elevated rates among students and young users, exacerbated by post-COVID increases in screen time for education and recreation, including video gaming (2, 3).

While mechanisms of DES are well described, there is limited synthesis of what interventions are actually applied and effective in real-world adolescent settings. A disconnect exists between mechanistic understanding and practical prevention strategies used in schools and daily life, leading to increased calls for objective assessment (4). Although the underlying mechanisms of DES—including ocular surface disruption, altered blink dynamics, and accommodative stress—have been extensively described in previous integrative and narrative reviews, current evidence remains largely focused on pathophysiological processes rather than practical mitigation. Consequently, there is a critical gap between mechanistic understanding and the implementation of effective, real-world prevention strategies.

Existing systematic reviews have examined DES prevalence, risk factors, and general management in broad populations or “young/pre-presbyopic” users (<40 years) (5, 6). One review of interventions for CVS management (45 RCTs) exists, but it is not age-restricted to adolescents (7). However, there remains a lack of systematic synthesis focusing on implemented and evaluated prevention strategies, particularly in adolescent populations where digital exposure patterns and behavioral factors differ substantially. Adolescents (typically 10–19 years per WHO definition) represent a particularly vulnerable group due to sustained exposure to educational and recreational screen activities, often combined with suboptimal visual habits and environmental conditions (8).

While mechanisms of DES are well described, there is limited synthesis of what interventions are applied and effective in real-world adolescent settings. A disconnect exists between mechanistic understanding and practical prevention strategies used in schools and daily life. To address this gap, the present systematic review aims to synthesize and critically appraise the evidence on applied prevention strategies specifically implemented in adolescent populations, with a focus on their effectiveness, feasibility, and real-world applicability across diverse settings.

### 1.2 Objectives

#### 1.2.1 Primary objective

To systematically identify, appraise, and synthesize published evidence on the types, implementation, and effectiveness of applied prevention strategies for reducing or preventing digital eye strain in adolescents.

#### 1.2.2 Secondary objectives

- To categorize prevention strategies (behavioral, environmental/ergonomic, optical, technological, educational/multicomponent).
- To evaluate their impact on symptom severity, prevalence, and objective ocular measures.
- To assess acceptability, compliance, and any adverse effects.
- To explore subgroup differences (e.g., by age, sex, country income level, baseline screen time).

## 2 Materials and methods

### 2.1 Study design and protocol registration

This study was conducted as a systematic review of applied prevention strategies for digital eye strain (DES) in adolescents. The review protocol was registered in the International Prospective Register of Systematic Reviews (PROSPERO) (9) prior to data extraction (CRD420261354954). The conduct and reporting of the review followed the Preferred Reporting Items for Systematic Reviews and Meta-Analyses (PRISMA 2020) (10) guidelines, and the protocol was developed in accordance with PRISMA-P recommendations. The research question was formulated using the PICOS (Population, Intervention, Comparison, Outcomes, and Study design) framework (11) and is presented in Table 1.

**Table 1:** Research question (PICOS framework).

Element	Description
Population (P)	Adolescents aged 10–19 years (or studies defining participants as “adolescents,” “teenagers,” “middle/high school students,” or providing a mean/median age within 10–19 with ≥80% of sample in this range). Screen users in any setting (school, home, leisure). Studies with mixed ages will be included only if adolescent subgroup data are extractable.
Intervention (I)	Any applied prevention strategy explicitly implemented to reduce/prevent DES/CVS symptoms. Examples: 20–20-20 rule or other break regimens; blink training; ergonomic adjustments (screen distance, posture, lighting); blue-light filtering lenses/filters/software; anti-glare screens; artificial tears or lubricating drops; educational programs/apps; multicomponent interventions; screen-time reduction programs. Must involve active application (not merely advice).
Comparison (C)	No intervention, usual care, placebo (e.g., clear lenses), or alternative prevention strategy.
Outcomes (O)	Primary: Change in DES/CVS symptoms (validated tools preferred, e.g., Computer Vision Syndrome Questionnaire [CVS-Q], Digital Eye Strain Questionnaire, or similar symptom scores); symptom prevalence or incidence. Secondary: Objective measures (tear break-up time, blink rate, accommodative amplitude, ocular surface staining); compliance/adherence; adverse events; quality-of-life or academic impact; cost or feasibility (if reported).
Study design (S)	Randomized controlled trials (RCTs), cluster-RCTs, quasi-experimental (non-randomized controlled), before-after/pre-post studies, and prospective cohort studies that evaluate an intervention. Cross-sectional studies will be excluded unless they include a clear intervention component with outcome assessment.

### 2.2 Information sources and search strategy

A comprehensive literature search was conducted in the following electronic databases: MEDLINE (via PubMed), Embase, Cochrane Central Register of Controlled Trials (CENTRAL), Scopus, Web of Science, CINAHL, and PsycINFO. Grey literature sources included Google Scholar (first 200 results), ProQuest Dissertations and Theses, OpenGrey, and clinical trial registries (ClinicalTrials.gov and WHO International Clinical Trials Registry Platform) (see Table 2).

**Table 2:** Included studies on adolescents.

Study	Type of intervention	Subjects	Results
Dos Santos et al. (2021) (15)	Behavioral/educational (multicomponent school-based screen-time reduction program)	*n* = 921 adolescents (10–16 yrs) in schools; cluster RCT over one academic year	The intervention showed minimal overall effect on screen time, with significant reduction only in TV viewing among a subgroup of students
Ryu et al. (2021) (16)	Optical (myopia-control spectacle lenses—DIMS vs. single vision)	*n* = 42 (22 adolescents, mean ~14.6 yrs.; 20 adults, mean ~24.9 yrs); experimental within-subject comparison during visual task	DIMS lenses significantly reduced subjective eye fatigue (~23% overall) compared with single-vision lenses in both adolescents and adults without affecting performance.
Chidi-Egboka et al. (2023) (17)	Behavioral/exposure (controlled smartphone use vs. rest)	n ≈ 36 children (school age)	Prolonged smartphone use induced dry eye signs and reduced tear stability, which improved after cessation, supporting screen reduction as an effective intervention
Hecht et al. (2025) (18)	Nutritional (oral astaxanthin vs. placebo)	*n* = 64 children/adolescents (10–14 yrs); randomized double-blind placebo-controlled trial, 84 days	Astaxanthin significantly reduced digital eye strain symptoms (~20%) and improved visual fatigue and tear-related parameters compared with placebo.
Mahalakshmi et al. (2025) (19)	Educational (video-assisted teaching program on ocular exercises and CVS prevention)	*n* = 60 adolescents (13–18 yrs); one-group pre–post experimental design in school setting	Video-assisted education significantly improved knowledge about ocular exercises and CVS prevention (adequate knowledge increased from 0 to 86.7%), though clinical outcomes were not assessed.
Soundari et al. (2025) (20)	Behavioral/vision therapy (ocular exercises + glasses vs. glasses only)	*n* = 170 schoolchildren (8–15 yrs.; 85 intervention, 85 control); randomized experimental study, 6 weeks	Ocular exercises significantly reduced eye strain and improved visual acuity with modest reduction in refractive error (~ − 0.5 D), unlike the control group.

The search strategy combined terms related to digital eye strain (e.g., “digital eye strain,” “computer vision syndrome,” “asthenopia”), adolescent populations (e.g., “adolescent,” “teen,” “school-aged”), and prevention or intervention strategies (e.g., “prevention,” “intervention,” “20–20-20 rule,” “blue light,” “ergonomics”). Controlled vocabulary (e.g., MeSH and Emtree terms) and free-text keywords were used, and search strategies were adapted for each database. Reference lists of included studies and relevant reviews were manually screened to identify additional eligible studies.

### 2.3 Eligibility criteria

The present review aimed to identify interventions for digital eye strain (DES) in adolescent populations. Adolescents were defined as individuals under 18 years of age. However, due to the limited number of studies conducted exclusively in adolescents, studies involving young adults (≥18 years) were also considered where relevant.

This approach was adopted to provide a more comprehensive synthesis of available evidence, as young adults share similar visual demands, screen-use behaviors, and underlying mechanisms of digital eye strain with adolescents. These include prolonged near work, reduced blink rate, tear film instability, and accommodative stress associated with digital device use.

Studies were included if they evaluated an intervention targeting digital eye strain symptoms, ocular surface function, or visual performance in populations that included adolescents and/or young adults.

### 2.4 Study selection

**Figure 1:**
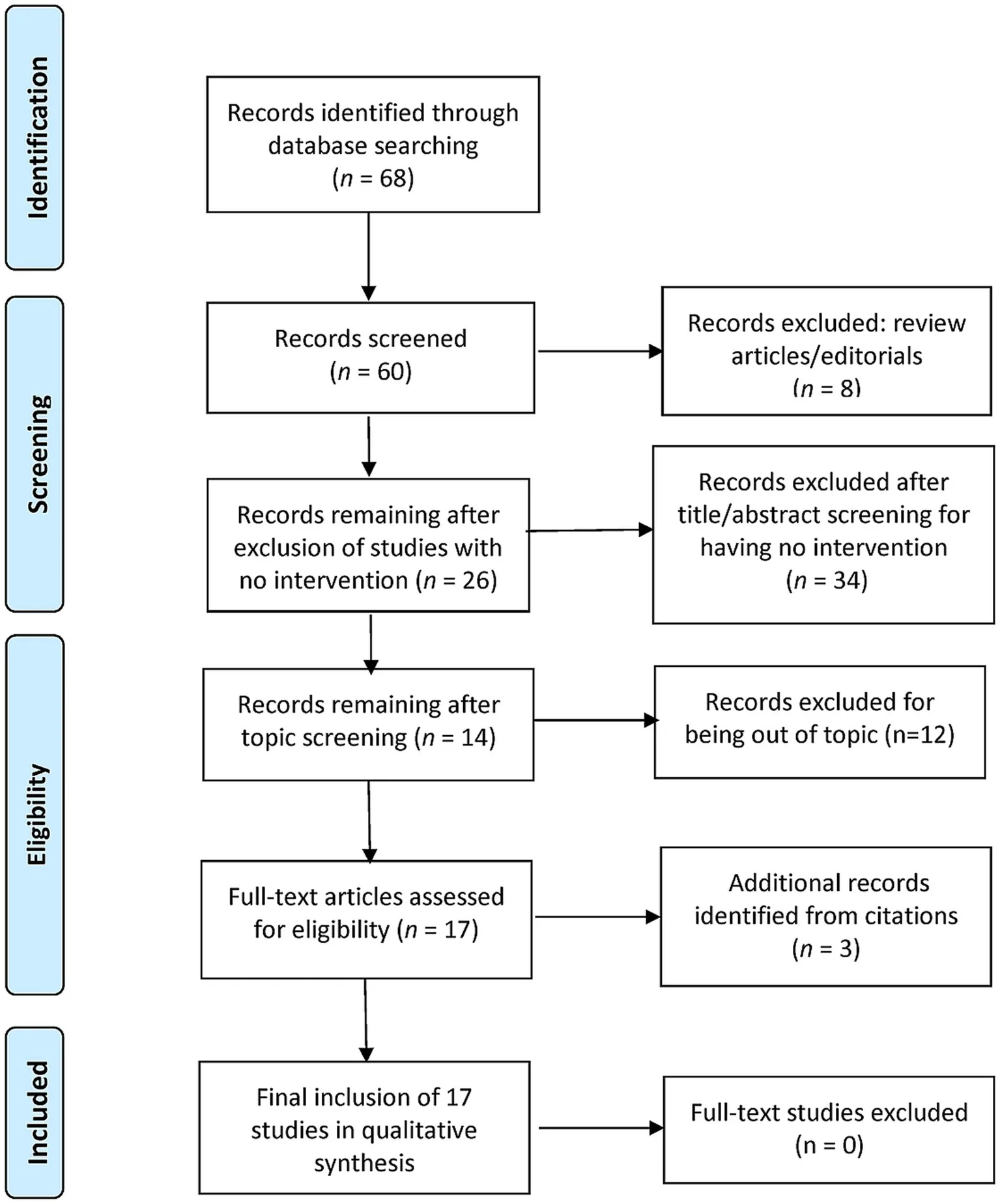
PRISMA flow diagram of the study selection process.

All identified records were imported into reference management software, and duplicates were removed. Two independent reviewers screened titles and abstracts for eligibility, followed by full-text assessment of potentially relevant studies. Disagreements at any stage were resolved through discussion or consultation with a third reviewer if necessary. The study selection process was documented using a PRISMA flow diagram (Figure 1). Fourteen articles were selected for inclusion out of sixty-eight initially identified, with three articles being added from references in those fourteen, bringing the total to seventeen.

### 2.5 Data extraction

Data were extracted independently by two reviewers using a standardized and piloted data extraction form. Prior to formal data extraction, the form was piloted on a sample of three included studies representing different study designs and intervention categories to assess completeness, clarity, and consistency. Minor modifications were made following the pilot phase to improve standardization of data recording.

Extracted data included study characteristics (authors, year, country, design, sample size, participant demographics), details of the intervention (type, duration, frequency, delivery method), comparator, outcome measures (including tools and time points), and key findings. Information on adherence, feasibility, and adverse events was also collected, where available.

Data extraction was performed independently by both reviewers. Any discrepancies were resolved through discussion and consensus, with re-examination of the original publication where necessary. No unresolved disagreements required third-party adjudication.

Risk of bias was assessed using the revised Cochrane Risk of Bias tool for randomized trials (RoB 2) (12) and the Risk Of Bias In Non-randomized Studies of Interventions (ROBINS-I) tool (13), while the certainty of evidence was evaluated using the Grading of Recommendations Assessment, Development and Evaluation (GRADE) approach (14).

## 3 Results

### 3.1 Rationale of expansion to include young adults

As evident in the PRISMA flow chart (Figure 1), only a limited number of studies were identified that exclusively investigated interventions for digital eye strain in adolescent populations. Most of the available evidence derives from studies conducted on young adults, with fewer trials including mixed populations or school-aged participants. Where adolescent-specific data were available, they were analyzed separately. Evidence from young adult populations was included to supplement the sparse adolescent literature, given the overlap in exposure patterns and physiological mechanisms underpinning digital eye strain.

### 3.2 Studies including only adolescents

The earliest study is the one by dos Santos et al. (15). This cluster-randomized school intervention (Movimente trial) targeted screen-time reduction through education, teacher training, and environmental modification in adolescents. While primarily behavioral, it addresses DES risk indirectly by reducing exposure. Results showed limited effect overall, with significant reduction only in TV time in a subgroup, highlighting difficulty in modifying screen habits in adolescents.

On the same year Ryu et al. (16) compared defocus incorporated multiple segment (DIMS) myopia-correcting spectacle lenses to standard single-vision lenses during a visual search task with an experimental design. The intervention targeted accommodative demand and visual processing load. Results showed that DIMS lenses significantly reduced subjective eye fatigue (~23% overall) in both adolescents and adults without impairing task performance, suggesting an accommodative or attentional mechanism reducing strain.

A prospective interventional study by Chidi-Egboka et al. (17) exposed children to prolonged smartphone use and measured changes in blink rate, tear function, and symptoms. While not therapeutic, it provides reversible intervention evidence showing that short-term discontinuation/rest improves tear parameters, supporting behavioral recommendations (breaks/reduction).

Hecht et al. (18) carried out on 2025 a randomized, double-blind placebo-controlled trial which evaluated oral astaxanthin (4 mg/day) in school-aged children (10–14 yrs) with ≥4 h/day screen use and DES symptoms. The intervention targeted oxidative stress and accommodative fatigue. Results showed significant reductions in CVS symptom scores (~20% improvement) and improvements in visual fatigue, tear production, and stereopsis, indicating both subjective and some functional benefits. However, these findings should be interpreted cautiously, as they derive from a single randomized trial with a relatively modest sample size, short follow-up duration, and no independent replication to date; furthermore, industry funding may introduce the potential for sponsorship bias.

Mahalakshmi et al. (19) on 2025 evaluated a video-assisted educational intervention (VATP) designed to improve adolescents’ knowledge about ocular exercises and preventive strategies for computer vision syndrome (CVS). The intervention delivered audiovisual instruction on eye exercises, ergonomics, and screen-use practices. Results showed a marked improvement in knowledge after the program, with most participants achieving adequate understanding, highlighting education as a strategy to support adoption of preventive behaviors rather than directly measuring symptom or performance outcomes.

Finally, Soundari et al. (20) carried out a randomized experimental study which evaluated the effectiveness of ocular exercises combined with regular spectacle use as an intervention to reduce eye strain and refractive error in children. Over a 6-week period, the intervention group performed structured eye exercises, resulting in significant reductions in eye strain, improvement in visual acuity, and a measurable reduction in refractive error (~ − 0.5 D) compared to controls (glasses only).

Table 3 presents a structured risk-of-bias (RoB) assessment for the studies on adolescents, using principles from RoB 2 (RCTs) and ROBINS-I/general appraisal frameworks for non-randomized or quasi-experimental studies.

**Table 3:** Risk of bias assessment for the studies of adolescents.

Study	Design	Selection bias	Performance/Blinding	Measurement bias	Attrition bias	Overall risk	Other concerns
Dos Santos et al. (2021) (15)	Cluster RCT	Moderate (cluster imbalance risk)	High (no blinding)	Moderate (self-reported outcomes)	Moderate	Moderate–high	Cluster design with potential contamination between groups; outcomes influenced by school—level implementation factors
Ryu et al. (2021) (16)	Experimental crossover	Low	Moderate (masking unclear)	Moderate (subjective fatigue outcome)	Low	Moderate	Small crossover study with limited external validity and short assessment period
Chidi-Egboka et al. (2023) (17)	Observational/intervention mix	High	High	High (self-report dominant)	Moderate	High	Mixed observational/intervention design with unclear intervention fidelity and limited causal inference
Hecht et al. (2025) (18)	RCT (double-blind)	Low	Low (double-blind)	Low–Moderate	Low	Low	Industry-funded nutritional intervention; single trial; no independent replication available
Mahalakshmi et al. (2025) (19)	Pre–post single group	High	High (no blinding)	Moderate (knowledge-based outcome)	Low–Moderate	High	Single-group pre-post design without control group; vulnerable to secular trends and Hawthorne effects
Soundari et al. (2025) (20)	Randomized experimental	Moderate (allocation clarity unclear)	High (no blinding)	Moderate–high (subjective + investigator involvement)	Low	Moderate–high	Incomplete reporting of randomization procedures and potential investigator involvement in intervention delivery

**Figure 2:**
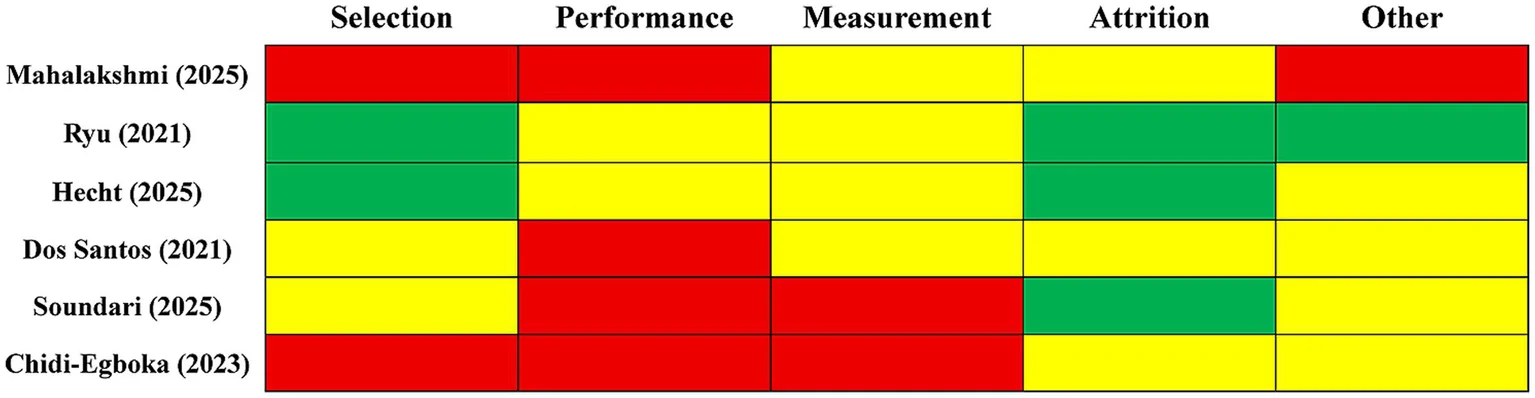
Risk of bias assessment of included adolescent studies. Green indicates low risk of bias, yellow indicates some concerns, and red indicates high risk of bias. Assessments were based on selection bias, performance bias, measurement bias, attrition bias, and additional study-specific concerns. The latter domain was included to capture issues such as industry sponsorship, lack of replication, single-arm designs, and other methodological limitations not fully reflected in standard bias domains. Overall risk-of-bias assessments informed the GRADE certainty-of-evidence evaluations presented in Tables 6, 7.

The methodological quality of the included studies varied considerably. Only one study employed a robust randomized double-blind design and was judged to have a low risk of bias, whereas several studies demonstrated moderate to high risk due to lack of blinding, reliance on subjective outcomes, and absence of appropriate control groups. These limitations should be considered when interpreting the reported effectiveness of interventions. Figure 2 presents the risk of bias assessment graphically.

### 3.3 Studies including young adults

Due to the limited number of intervention studies conducted exclusively in adolescent populations, studies involving young adults were also considered where relevant. This approach is justified by the substantial overlap in visual demands, screen-use behaviors, and underlying mechanisms of digital eye strain between adolescents and young adults. However, the extrapolation of findings should be interpreted with caution, as age-related differences in ocular development and behavior may influence treatment response.

The prospective observational study study by Bogdănici et al. (21) was carried out in 2017 and primarily examined the impact of digital device use on vision and Computer Vision Syndrome (CVS), but also noted artificial tears as a symptomatic intervention. The findings indicate that instillation of artificial tear drops can improve visual comfort in individuals with dry eye symptoms associated with device use, addressing ocular surface dryness rather than underlying visual performance deficits.

Yammouni and Evans (22) on 2020 investigated low-powered convex “add” lenses (+0.50D, +0.75D, +1.25D) as an intervention for digital eye strain (DES) in pre-presbyopic adults. Using a double-masked, randomized cross-over design, participants viewed text under different lens conditions while reading performance (Wilkins Rate of Reading Test) and subjective preference were assessed. The intervention aims to reduce accommodative demand during digital device use. Results showed that low adds, particularly +0.50D and +0.75D (most commonly +0.75D), improved reading performance and were preferred by many users, with about one-quarter achieving >10% faster reading; +1.25D showed no significant benefit.

Bhargava et al. (23) presented on 2023 results from a large randomized controlled trial that evaluated oral omega-3 fatty acid supplementation (EPA + DHA) in visual display terminal (VDT) users with dry eye over 6 months. The intervention significantly improved dry eye symptoms, tear film stability (TBUT), tear production (Schirmer), and reduced tear osmolarity, compared with placebo. Effects were more pronounced in individuals with low baseline omega-3 levels, although prior literature shows mixed findings across populations.

On the same year Trancoso Vaz et al. (24) published the results of a prospective controlled study that evaluated preservative-free hyaluronic acid artificial tears (0.15%) combined with ergo-ophthalmological measures (e.g., brief breaks and visual hygiene) in individuals exposed to intensive screen use during video gaming. Compared with ergonomic measures alone, the combined intervention prevented worsening of dry eye symptoms and significantly reduced ocular fatigue and dryness over a 3-day gaming period, although objective clinical signs showed minimal short-term change.

Tu and Pang (25) presented an abstract at the 2023 Association for Research in Vision and Ophthalmology (ARVO) Annual Meeting (2023, ARVO abstract) results from a controlled experimental study in young adults (mean ~26 yrs) manipulated blink rate and visual ergonomics (working distance + lighting) during screen viewing. While higher blink rate alone did not reduce symptoms, optimal viewing conditions (longer distance, adequate lighting) significantly reduced multiple digital eye strain symptoms (dryness, eyestrain, discomfort), highlighting ergonomics as the key mechanism.

Lin et al. (26) published on 2024 results from an experimental visual ergonomics study that evaluated display characteristics (contrast polarity and luminance levels) of a desktop head-up display (HUD) as an indirect intervention to reduce digital eye strain. The intervention involved optimizing text polarity (positive vs. negative) and screen luminance (20–200 cd/m^2^) under office lighting conditions (300 lx). Results showed that positive polarity (dark text on light background) reduced visual fatigue and discomfort, and that visual fatigue followed an inverted-U relationship with luminance, with optimal comfort around ~150–153 cd/m^2^, where fatigue and discomfort were minimized while performance peaked.

Lopresti and Smith (27) carried out a randomized, double-blind placebo-controlled trial that examined lutein + zeaxanthin supplementation in high screen users (many young adults). The intervention targets retinal and tear-film physiology. Results showed improvements in objective ocular measures (tear production, tear film stability, photo-stress recovery), though subjective eye strain improvements were not significantly different vs. placebo.

Redondo et al. (28) run an experimental study that evaluated different break schedules during prolonged near work (no break, one break at 20 min—20-20-20 rule, breaks every 10 min, and self-paced breaks) as a behavioral intervention for digital eye strain (DES). During a 40-min reading task, more frequent (every 10 min) or self-paced breaks reduced symptoms such as eye irritation and eyestrain, stabilized accommodative variability, and reduced near work-induced transient myopia, whereas the commonly recommended 20–20-20 rule showed limited benefit compared to no break.

Watcharapalakorn et al. (29) published the results of a randomized crossover study that evaluated the benefit of blue-filtering spectacle lenses over standard lenses (anti-reflective and uncoated CR39) during a 120-min digital reading task on the reduction of blue light exposure from screens. Results showed that, although digital device use increased dry eye symptoms and worsened tear film metrics, blue-filtering lenses did not significantly improve symptoms or tear film stability compared with control lenses, indicating no short-term benefit for digital eye strain or dry eye.

Xu et al. (30) carried out a prospective randomized digital intervention which evaluated a smartphone-based blink training application to improve blink dynamics and tear film stability in screen users (primarily young, heavy digital users). The intervention aimed to correct reduced blink frequency/incomplete blinking, a major DES mechanism. Results showed improved blink behavior and ocular surface stability, with reduced dry-eye–related symptoms.

Finally, Ortiz-Toquero et al. (31) published on 2026 results from a prospective single-arm study that evaluated preservative-free artificial tears (Systane Ultra UD) used four times daily for one month in digital device users with digital eye strain (DES) and dryness symptoms. The intervention targeted ocular surface lubrication and tear volume enhancement to reduce dryness-related discomfort. Results showed significant reductions in subjective DES and dry eye symptoms, but no meaningful improvements in objective visual task performance (e.g., reading speed, blink rate) or tear film stability measures.

Table 4 briefly outlines the aforementioned studies while Table 5 presents a structured risk-of-bias (RoB) assessment for the studies on young adults, using principles from RoB 2 (RCTs) and ROBINS-I/general appraisal frameworks for non-randomized or quasi-experimental studies. The ‘Other concerns’ section represents limitations not fully captured by standard bias domains, including industry sponsorship, lack of independent replication, conference-only publication status, uncontrolled study designs, uncertainty regarding intervention fidelity, and limitations affecting real-world applicability and external validity.

**Table 4:** Studies including young adults.

Study	Type of intervention	Subjects	Results
Bogdănici et al. (2017) (21)	Supportive/symptomatic (artificial tears for dry eye relief)	*n* = 60 (30 schoolchildren, mean age ~11.9; 30 young adults/patients, mean age ~21.4); observational study of digital device users	Artificial tears were reported to improve visual comfort in individuals with dry eye symptoms related to digital device use, though no controlled effect size or performance outcome was quantified
Yammouni and Evans (2020) (22)	Optical (low-power convex “add” spectacle lenses: +0.50D, +0.75D, +1.25D vs. plano control)	*n* = 107 pre-presbyopic adults (20–40 yrs) with DES symptoms; majority heavy computer users (~8 h/day), double-masked cross-over trial	Low adds (+0.50D and +0.75D, especially +0.75D) significantly improved reading performance and were preferred by many participants, with ~24% reading >10% faster, while +1.25D showed no significant benefit vs. control
Bhargava et al. (2023) (23)	Nutritional/pharmacological (oral omega-3 fatty acid supplementation vs. placebo)	*n* = 950 VDT users with dry eye (470 intervention, 480 placebo); randomized controlled trial, 6-month follow-up	Omega-3 supplementation significantly improved dry eye symptoms and tear film parameters compared with placebo, especially in individuals with low baseline omega-3 index.
Trancoso Vaz et al. (2023) (24)	Combined (pharmacological + behavioral: hyaluronic acid artificial tears 4×/day + ergophthalmological measures)	*n* = 56 adult gamers (≥18 yrs.; ≥6 h/day gaming for 3 days); randomized controlled study (SG: *n* = 30 vs. CG: *n* = 26)	Artificial tears plus ergonomic measures prevented symptom worsening and significantly reduced ocular fatigue and dryness compared to ergonomic measures alone during intensive screen use.
Tu and Pang (2023, ARVO abstract) (25)	Behavioral/ergonomic (blink rate manipulation + viewing distance/lighting conditions)	*n* = 38 young adults (mean 25.9 yrs.; 22–34); randomized repeated-measures lab experiment	Optimal viewing distance (60 cm) and proper lighting significantly reduced DES symptoms, whereas increasing blink rate alone did not produce significant benefit.
Lin et al. (2024) (26)	Ergonomic/display optimization (contrast polarity and luminance adjustment in HUD display)	*n* = 36 (polarity experiment) and *n* = 21 (luminance experiment); healthy young adults; controlled laboratory study	Positive contrast polarity and an optimal luminance of ~150–153 cd/m^2^ significantly reduced visual fatigue and discomfort while maintaining best performance under office lighting.
Lopresti and Smith (2025) (27)	Nutritional (lutein 10 mg + zeaxanthin 2 mg daily vs. placebo)	*n* = 70 high screen users (18–65 yrs.; includes young adults subgroup), ≥6 h/day screen use; RCT, 6 months	Supplementation improved objective tear and visual function measures but did not significantly improve subjective digital eye strain compared with placebo.
Redondo et al. (2025) (28)	Behavioral/ergonomic (break schedules during screen use: none vs. 20–20-20 vs. every 10 min vs. self-paced breaks)	*n* = 24 healthy young adults (mean age ~21 years); repeated-measures experimental study with 4 randomized conditions	Frequent (every 10 min) or self-paced breaks reduced DES symptoms and accommodative instability compared to no break or the 20–20-20 rule, which showed minimal benefit.
Watcharapalakorn et al. (2025) (29)	Optical (blue-filtering spectacle lenses vs. anti-reflective and uncoated lenses)	*n* = 26 healthy young adults (mean age ~21.7 yrs); randomized crossover study with 3 lens conditions and repeated digital tasks	Blue-filtering lenses did not significantly reduce dry eye symptoms or improve tear film parameters compared with standard lenses during digital screen use.
Xu et al. (2025) (30)	Digital/behavioral (smartphone-based blink training)	young digital device users (exact *n* varies by cohort; mean age 24.15, prospective randomized study)	Blink training improved blinking patterns and tear film stability, reducing dry eye–related digital eye strain symptoms.
Ortiz-Toquero et al. (2026) (31)	Pharmacological (preservative-free artificial tears, 4×/day for 1 month)	*n* = 30 digital device users (18–55 yrs) with DES and dry eye symptoms, ≥4 h/day screen use; prospective single-arm study	Artificial tears significantly improved subjective DES and dry eye symptoms but did not significantly improve reading performance, blink behavior, or tear film stability.

**Table 5:** Risk of bias assessment for the studies involving young adults.

Study	Design	Selection bias	Performance/Blinding	Measurement bias	Attrition bias	Overall risk	Other concerns
Bogdănici et al. (2017) (21)	Observational	High	High	High (self-report dominant)	Moderate	High	Observational design limits causal inference; intervention exposure not well controlled
Yammouni and Evans (2020) (22)	Randomized crossover	Low	Moderate (masking limited)	Moderate (subjective reading outcomes)	Low	Moderate	Short-term crossover evaluation; limited generalizability to prolonged digital-device use
Bhargava et al. (2023) (23)	RCT (large)	Low	Moderate (blinding likely but not always clear)	Moderate (mixed outcomes)	Low	Low–Moderate	Single-center study; intervention adherence not extensively reported
Trancoso Vaz et al. (2023) (24)	Randomized controlled	Low	Moderate–high (masking uncertain)	Moderate	Low	Moderate	Limited follow-up duration; real-world sustainability of intervention uncertain
Tu and Pang (2023) (25)	Experimental repeated measures	Low	High (no masking)	Moderate (self-reported DES scores)	Low	Moderate	Conference abstract only, limited methodological detail available
Lin et al. (2024) (26)	Experimental lab study	Moderate	High (no blinding)	Moderate (subjective + objective mix)	Low	Moderate	Laboratory-based environment may limit ecological validity
Lopresti and Smith (2025) (27)	RCT (double-blind)	Low	Low	Moderate (subjective outcomes inconsistent)	Low	Low	Nutritional intervention with potential commercial/supplement-industry involvement; replication limited
Redondo et al. (2025) (28)	Randomized experimental	Low	Moderate (partial masking)	Moderate (symptom-based outcomes)	Low	Moderate	Behavioral intervention dependent on participant adherence; adherence reporting incomplete
Watcharapalakorn et al. (2025) (29)	Randomized crossover	Low	Moderate	Moderate–Low (objective tear metrics)	Low	Moderate	Short intervention period; long-term effectiveness unknown
Xu et al. (2025) (30)	Likely experimental/observational	Moderate–high	High	Moderate–high	Moderate	Moderate–high	Intervention methodology incompletely described; uncertain reproducibility
Ortiz- Toquero (2026) (31)	Prospective single-arm	High (no control)	High	Moderate (subjective + objective mismatch)	Low	High	Single-arm prospective design without comparator group; substantial risk of confounding

**Figure 3:**
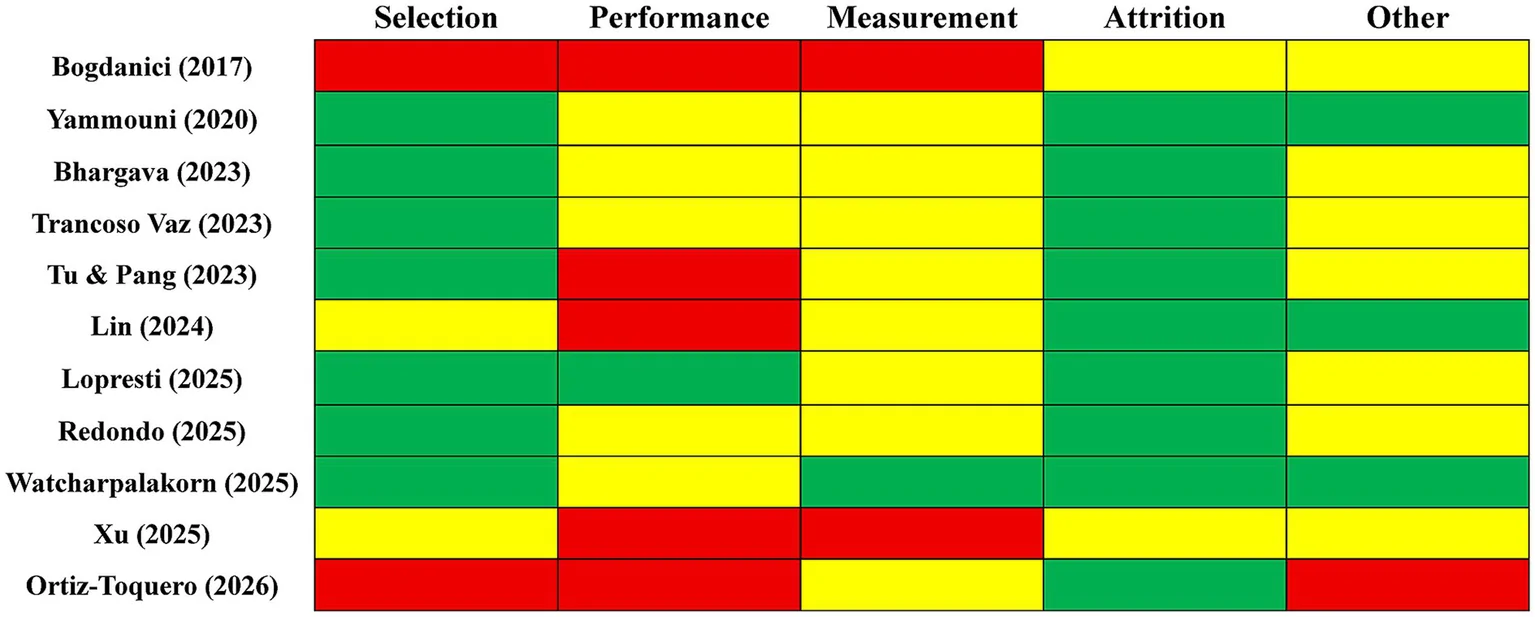
Risk of bias assessment of included studies on young adults. Risk-of-bias summary of included studies. Green indicates low risk of bias, yellow indicates some concerns, and red indicates high risk of bias. Assessments were based on selection bias, performance bias, measurement bias, attrition bias, and additional study-specific concerns. The latter domain was included to capture issues such as industry sponsorship, lack of replication, single-arm designs, and other methodological limitations not fully reflected in standard bias domains. Overall risk-of-bias assessments informed the GRADE certainty-of-evidence evaluations presented in Tables 6, 7.

Risk-of-bias assessment of studies involving young adults demonstrated generally higher methodological quality than studies conducted exclusively in adolescents. Several randomized controlled trials showed a low risk of bias across most domains, whereas observational studies and uncontrolled designs were associated with a higher risk of bias. The most common methodological concerns included incomplete masking, reliance on subjective symptom-based outcomes, limited follow-up, and issues relating to external validity or intervention adherence. Figures 2, 3 provide graphical summaries of the risk-of-bias assessments. Overall, two studies were classified as low risk of bias, six studies as having some concerns, and three studies as high risk of bias.

### 3.4 Overall certainty of evidence

Using the GRADE framework, the overall certainty of evidence was judged to be low. This was primarily due to methodological limitations across studies, inconsistency of findings, and indirectness arising from reliance on young adult populations rather than adolescents. Tables 6, 7 present the evidence profile and the overall rating by outcome domain and population. The certainty of evidence was generally low because of indirectness arising from reliance on young-adult populations, heterogeneity in intervention protocols and outcome measures, and the small number of adolescent-specific trials. Consequently, the findings should be interpreted as indicative rather than definitive and should not be considered sufficient for guideline-level recommendations.

**Table 6:** GRADE evidence profile—overall rating by outcome domain for adolescents only.

Outcome	Study types	Certainty (GRADE)	Key reasons for downgrading
Reduction in DES symptoms (subjective)	RCT + experimental + observational	Low ⬇⬇	Risk of bias (lack of blinding, subjective outcomes), imprecision (small sample sizes), inconsistency across intervention types
Behavioral/ergonomic intervention	Experimental studies	Low ⬇⬇	Small number of studies, risk of bias from self-reported outcomes, imprecision due to limited sample sizes
Nutritional	High-quality RCT + others	Low ⬇⬇	Imprecision (single adolescent RCT), indirectness of outcome measures, lack of independent replication, potential concern regarding sponsorship bias
Educational interventions	Pre–post designs	Very low ⬇⬇⬇	High risk of bias, uncontrolled pre-post designs, imprecision, limited clinical outcome assessment
Overall adolescent evidence base		Low ⬇⬇	Limited number of adolescent-specific studies, imprecision, heterogeneity of interventions and outcomes

The adolescent evidence base was downgraded primarily because few intervention studies were conducted exclusively in adolescents, and many studies relied on subjective symptom outcomes, small samples, or non-randomized designs.

**Table 7:** GRADE evidence profile—overall rating by outcome domain for young adults only.

Outcome	Study types	Certainty (GRADE)	Key reasons for downgrading
Reduction in DES symptoms (subjective)	RCT + experimental + observational	Low ⬇⬇	Indirectness (population differs from review target), inconsistency across interventions and symptom measures
Objective ocular surface outcome	RCT + experimental	Moderate ⬇	Indirectness due to reliance on young-adult populations rather than adolescents
Behavioral/ergonomic intervention	Experimental studies	Low ⬇⬇	Indirectness + imprecision
Visual performance/reading outcome	Experimental studies	Low ⬇⬇	Inconsistent findings, indirectness, variability in study methodologies
Nutritional interventions	RCT + others	Low ⬇⬇	Indirectness, limited number of studies, possible publication bias, lack of long-term replication

Young-adult evidence was frequently downgraded for indirectness because the primary review question concerned adolescents. Although behavioral and physiological similarities support cautious extrapolation, findings from young-adult studies cannot be considered equivalent to adolescent-specific evidence.

## 4 Discussion

### 4.1 Overall findings

The findings of this review highlight a notable gap in literature, with relatively few intervention studies conducted specifically in adolescent populations. As a result, evidence from young adult populations was incorporated to provide a more complete understanding of potential management strategies for digital eye strain. Table 8 summarizes the distribution of intervention types in the included studies. Adolescent evidence was concentrated primarily in educational and behavioral interventions, whereas evidence for ocular-surface, optical, and nutritional approaches was limited. Several intervention categories, particularly ocular-surface management, were represented only by studies conducted in young-adult populations.

**Table 8:** Distribution of intervention types across included studies.

Intervention category	Adolescent studies	Young adult studies	Main OUTCOME
Behavioral	Soundari et al. (2025) (20)	Trancoso Vaz et al. (2023) (24) and Redondo et al. (2025) (28)	Reduced symptom scores
Ergonomic/Environmental	Soundari et al. (2025) (20)	Tu and Pang (2023) (25) and Lin et al. (2024) (26)	Reduced visual fatigue
Optical	Ryu et al. (2021) (16)	Yammouni and Evans (2020) (22) and Xu et al. (2025) (30)	Reduced visual fatigue, no significant improvement in performance
Ocular surface management	None	Bhargava et al. (2023) (23), Watcharapalakorn et al. (2025) (29), Ortiz-Toquero et al. (2026) (31)	Improved comfort and dryness symptoms
Nutritional	Hecht et al. (2025) (18)	Lopresti and Smith (2025) (27)	Preliminary benefit
Educational	Dos Santos et al. (2021) (15), Chidi-Egboka et al. (2023) (17), Mahalakshmi et al. (2025) (19)	Bogdănici et al. (2017) (21)	Improved awareness, limited clinical outcomes

The available adolescent-focused evidence can be broadly categorized into nutritional interventions, behavioral/school-based interventions, and exposure studies examining the physiological effects of digital device use. Among these, evidence supporting astaxanthin derives primarily from a single randomized trial and therefore should be considered preliminary pending independent replication, while behavioral and school-based interventions demonstrate modest but potentially meaningful benefits. Exposure studies further support the biological plausibility of preventive strategies by demonstrating adverse ocular effects associated with intensive smartphone use.

### 4.2 Effectiveness versus real-world adoption

A key finding of this review is the apparent disconnect between the popularity of certain prevention strategies and the strength of supporting evidence. Interventions such as blue-light filtering lenses and screen filters are widely marketed and frequently adopted in clinical and consumer settings; however, their effectiveness in reducing DES symptoms appears inconsistent across studies. In contrast, low-cost behavioral strategies—such as scheduled breaks (e.g., the 20-20-20 rule), blink awareness training, and posture correction—demonstrated more reproducible benefits, despite receiving comparatively less emphasis in commercial or clinical practice.

This discrepancy highlights a critical issue in DES management: the influence of perception, accessibility, and market forces on intervention uptake, which may not align with evidence-based effectiveness. These findings underscore the need for clinicians and policymakers to prioritize interventions supported by empirical evidence and to address misconceptions regarding less effective but widely promoted strategies.

### 4.3 Adolescent-specific considerations

This review emphasizes the importance of considering adolescent-specific factors when evaluating and implementing DES prevention strategies. Adolescents differ from adult populations not only in terms of screen exposure duration but also in behavioral patterns, including multitasking, prolonged uninterrupted use, and frequent reliance on handheld devices (32). These patterns may exacerbate DES symptoms and influence the effectiveness of various interventions (5).

Our review has shown that important gaps remain when compared with the adult DES literature. Adolescent-specific trials evaluating optical interventions, artificial tears or pharmacological approaches, and structured ergonomic or break-schedule interventions are limited or absent, highlighting key priorities for future research. Overall, compared to young adults, the adolescent DES intervention evidence base is still underdeveloped. The reliance on young adult populations further limits the applicability of findings to adolescents. Although substantial overlap exists between adolescents and young adults with respect to screen exposure, visual demands, and several DES risk factors, evidence derived from young-adult populations should be regarded as indirect evidence and not as a substitute for adolescent-specific intervention studies. Both groups are characterized by high levels of digital device use, prolonged near viewing distances, and modifiable risk factors such as reduced blink rate and suboptimal ergonomic conditions. Furthermore, the underlying mechanisms of digital eye strain (including accommodative stress, tear film instability, and visual fatigue), are largely consistent across these age groups. Nevertheless, caution is warranted when extrapolating findings from young adults to adolescents, as developmental differences in visual function, habits, and compliance with interventions may influence outcomes.

Several interventions relied on sustained participant engagement, including blink-training applications, scheduled break regimens, ergonomic recommendations, and regular use of artificial tears. Educational and school-based programs demonstrated that awareness and preventive behaviors can be promoted through structured environments; however, evidence from broader screen-time interventions suggests that modifying habitual digital behaviors remains challenging in adolescents (15, 17, 18). Behavioral patterns such as prolonged smartphone use, gaming, and uninterrupted screen engagement may reduce adherence to preventive recommendations in everyday settings. Consequently, the real-world effectiveness of DES prevention strategies appears to depend not only on their clinical efficacy but also on their feasibility, acceptability, and integration into adolescents’ educational and recreational environments. Similar compliance issues do exist in young adult populations (24, 28–31), yet their comparative impact was lower.

Furthermore, social and educational factors—including academic demands, remote learning environments, and recreational screen use—limit the feasibility of certain interventions, such as strict screen-time reduction. These findings indicate that prevention strategies in adolescents must be not only effective but also adaptable to real-life constraints and behavioral tendencies within this population (2).

Adherence data were reported inconsistently across studies. Where assessed, compliance appeared higher for structured school-based programmes and lower for self-directed behavioral interventions. Adverse events were infrequently reported and were generally mild or absent. The scarcity of adherence and acceptability data limited assessment of long-term sustainability and real-world implementation.

### 4.4 Implementation and feasibility

A major contribution of this review lies in its focus on implementation and real-world applicability. While many interventions demonstrate efficacy under controlled conditions, their scalability and sustainability in everyday settings remain uncertain (15, 18, 27). Behavioral interventions, although conceptually simple and low cost, rely heavily on user adherence and may require reinforcement mechanisms, such as reminders or integration into school routines (15, 19, 30).

Multicomponent interventions—combining behavioral guidance, ergonomic adjustments, and educational components—appear to offer a more pragmatic approach, as they address multiple contributing factors simultaneously (15, 24). These interventions may also improve adherence by embedding behavioral changes within structured environments, such as classrooms or organized digital platforms (15, 19, 30).

Technological solutions, including smartphone applications and digital prompts, offer a potentially scalable method for delivering behavioral interventions (30). However, evidence regarding their long-term effectiveness remains limited, and further research is needed to assess their role in sustained behavior modification (17, 28, 30).

Overall, the findings suggest that the most effective prevention strategies are those that balance efficacy with feasibility, requiring minimal effort while integrating seamlessly into daily routines (15, 17, 24, 30).

**Figure 4:**
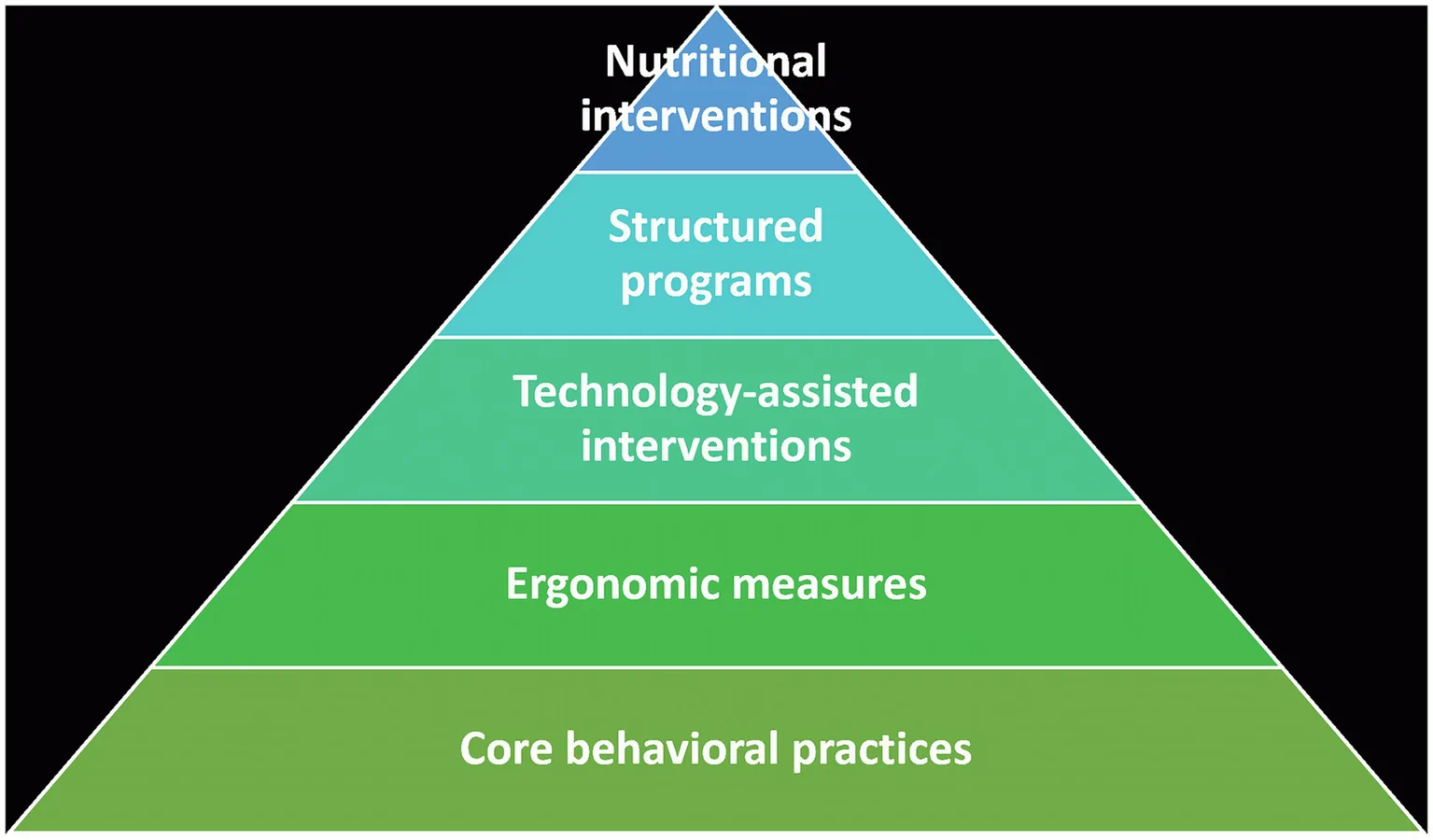
Proposed digital eye strain (DES) prevention pyramid for adolescents. This author-derived conceptual framework is intended to facilitate interpretation of the reviewed literature from an implementation and scalability perspective and should not be interpreted as a quantitative ranking of effectiveness or certainty of evidence. Prevention strategies are arranged according to their estimated implementation burden, resource requirements, and potential population scalability. Foundational behavioral and ergonomic measures occupy the base of the pyramid because they are generally low cost, easily implemented, and broadly applicable across school and home environments. Technology-assisted interventions and structured educational or school-based programmes occupy intermediate levels, reflecting their greater organisational requirements but potential to reinforce behavioral change. Nutritional interventions are positioned at the apex because implementation typically depends on sustained adherence, ongoing individual engagement, and additional cost considerations. The framework is intended as a heuristic tool to support discussion of implementation and feasibility rather than as a direct finding of the systematic review.

To facilitate interpretation of the evidence, we propose an author-derived conceptual framework, the DES Prevention Pyramid (Figure 4), which organizes interventions according to their apparent scalability, implementation burden, and resource requirements based on the studies reviewed. This framework should be regarded as an interpretive tool intended to guide discussion rather than a direct finding of the systematic review. At the base of the pyramid are low-cost behavioral and ergonomic measures, including regular blinking, scheduled screen breaks, appropriate viewing distance, and environmental adjustments. These strategies require minimal resources and can be implemented broadly across school and home settings. Intermediate levels include technology-assisted interventions, such as blink-training applications and digital reminder systems, as well as structured educational and school-based programs that provide behavioral reinforcement. At the apex are nutritional interventions, which may demonstrate clinical benefit but require ongoing adherence, greater individual commitment, and potentially higher costs. This framework suggests that the most sustainable public health approaches may be those positioned at the lower levels of the pyramid, where scalability and feasibility are greatest, while higher-level interventions may serve as adjunctive strategies for selected individuals.

### 4.5 Mechanistic context

Although this review did not aim to provide an in-depth analysis of pathophysiological mechanisms, the observed effectiveness of behavioral and ergonomic interventions is consistent with established understanding of DES (4, 6). Reduced blink rate, increased interblink interval, and sustained accommodative demand during prolonged screen use are known contributors to ocular surface instability and visual discomfort (4, 17). Interventions that promote regular breaks, increase blink frequency, and optimize viewing conditions likely mitigate these mechanisms, thereby reducing symptom burden (16, 24, 28, 30).

Importantly, this review extends beyond mechanistic explanations by linking these physiological processes to practical, implementable strategies, thereby bridging the gap between theoretical understanding and clinical application (15, 19, 30).

### 4.6 Limitations of the evidence base

This review has several limitations. First, there is a paucity of high-quality studies conducted exclusively in adolescent populations, which limits the ability to draw age-specific conclusions. Second, the inclusion of studies involving young adults introduces heterogeneity in the study populations, which may affect the generalizability of findings to younger individuals.

Although the inclusion of young adult studies was justified based on shared pathophysiological mechanisms and behavioral patterns, differences in ocular development, lifestyle factors, and adherence to interventions between adolescents and young adults should be considered when interpreting the results.

Various methodological hurdles limit the generalizability and reproducibility of the findings. First, there was considerable heterogeneity in outcome measures, with many studies using non-standardized or non-validated symptom assessment tools. This limits the comparability of results and may introduce measurement bias.

Second, sample sizes were often small, and study durations were relatively short, restricting the ability to assess long-term effectiveness and sustainability of interventions. Third, variability in intervention design, including differences in duration, intensity, and delivery methods—complicates synthesis and interpretation of results.

Additionally, the risk of bias across studies was variable, with several non-randomized designs and limited reporting of allocation methods and blinding. These methodological limitations highlight the need for more rigorously designed trials with standardized outcome measures and longer follow-up periods.

Future research should prioritize well-designed randomized controlled trials specifically targeting adolescent populations to better inform age-appropriate clinical recommendations.

### 4.7 Strengths of this review

This review has several notable strengths. It is, to our knowledge, the first systematic synthesis specifically focusing on applied prevention strategies for DES in adolescents, while explicitly incorporating supporting evidence from young-adult populations when adolescent-specific data were limited. This approach allowed identification of important evidence gaps and highlighted intervention categories requiring future adolescent-focused investigation. The review was conducted using a structured methodology in accordance with PRISMA guidelines, with predefined eligibility criteria, duplicate screening, standardized data extraction, formal risk-of-bias assessment, and GRADE-based evaluation of evidence certainty. In response to the limited adolescent evidence base, direct adolescent evidence and indirect supporting evidence from young-adult populations were evaluated separately, improving transparency regarding the applicability of findings.

An additional strength is the systematic appraisal of methodological quality. Risk-of-bias assessments identified important limitations across the literature, including lack of blinding, reliance on subjective symptom measures, small sample sizes, and limited replication of findings. These assessments informed the GRADE evaluations and ensured that conclusions were aligned with the strength of the underlying evidence rather than solely with study outcomes.

Finally, the categorization of interventions into behavioral, ergonomic, ocular-surface, optical, nutritional, educational, and multicomponent approaches provided a clinically meaningful framework for comparing prevention strategies and identifying areas in which evidence remains sparse. Although feasibility and adherence data were inconsistently reported, their consideration helped identify important gaps for future implementation-focused research.

### 4.8 Clinical and public health implications

The findings of this review should be interpreted considering the limited quantity and generally low certainty of the available adolescent-specific evidence. Nevertheless, the current literature suggests that behavioral and ergonomic interventions, including regular screen breaks, blink-awareness strategies, and appropriate screen positioning, may represent pragmatic and relatively low-risk approaches for managing digital eye strain in adolescents.

From a public health perspective, school-based educational programs and digital-health initiatives may provide useful platforms for promoting awareness of healthy screen-use practices. School-based interventions may offer an effective platform for delivering and reinforcing preventive strategies, particularly given their potential to improve adherence. Public health initiatives should also consider the development of standardized guidelines for digital device use in adolescents, integrating visual health alongside broader concerns such as mental well-being and physical activity. However, the effectiveness of such programs remains incompletely established, and further research is required to determine their long-term impact on symptom reduction and behavioral change.

Given the limited adolescent-specific evidence base, the development of formal clinical or public-health guidelines should be approached cautiously. Future recommendations should ideally be informed by adequately powered adolescent-focused intervention studies that evaluate not only efficacy, but also adherence, acceptability, feasibility, and long-term sustainability.

### 4.9 Future research directions

Future research should focus on addressing the methodological limitations identified in this review. There is a need for well-designed, adequately powered randomized controlled trials using validated and standardized DES outcome measures. Longitudinal studies are also required to assess the sustainability of intervention effects over time.

In addition, greater attention should be given to intervention adherence and behavioral factors, including strategies to improve compliance in adolescent populations. Technological solutions, such as digital reminders and adaptive applications, warrant further investigation as scalable tools for behavior modification.

Finally, research should move towards pragmatic, real-world study designs that reflect the complexity of adolescent digital environments, with an emphasis on interventions that are both effective and feasible at-scale.

## 5 Conclusion

In conclusion, this review highlights that effective prevention of digital eye strain in adolescents requires a shift from isolated or commercially driven interventions towards practical, behaviorally informed, and contextually feasible strategies. Bridging the gap between evidence and implementation will be essential to addressing the growing burden of DES in this high-risk population.

This review highlights a significant gap in the literature regarding interventions for digital eye strain in adolescents. Despite the high prevalence of DES in this population, relatively few studies have evaluated targeted interventions exclusively in individuals under 18 years of age, and the certainty of the available evidence remains low.

In the absence of sufficient adolescent-specific evidence, findings from studies conducted in young adults were incorporated to inform the synthesis. This approach is supported by substantial overlap between adolescents and young adults in terms of digital device usage patterns, visual demands, and underlying mechanisms of digital eye strain, including accommodative stress, tear film instability, and reduced blink rate. Across these populations, behavioral and ergonomic interventions, as well as ocular surface management strategies, consistently demonstrated the greatest potential for reducing symptoms. Still. evidence derived from young adults should be regarded as indirect rather than adolescent-specific evidence. Consequently, caution is warranted when extrapolating findings between these populations.

Across the included studies, behavioral and ergonomic interventions appeared to demonstrate the most consistent potential for reducing DES symptoms, whereas evidence supporting optical, nutritional, educational, and ocular-surface interventions was more limited or heterogeneous. However, the overall certainty of evidence ranged from ‘very low’ to ‘low’ for most adolescent-specific outcomes.

At present, the available evidence is insufficient to support definitive clinical recommendations for adolescents. Nevertheless, behavioral and ergonomic strategies represent low-cost, low-risk interventions that may be considered while awaiting stronger evidence. Future research should prioritize adequately powered, adolescent-specific randomized controlled trials evaluating intervention effectiveness, adherence, acceptability, and long-term implementation in real-world educational and recreational settings.
