# Supplementary material for: Interventions for digital eye strain in adolescents: a structured review with supporting evidence from young adult populations
Source: Front Public Health. 2026 Sep 10;14:1942125. doi: 10.3389/fpubh.2026.1942125 (PMC13602525; doi:10.3389/fpubh.2026.1942125)
Supplement: Supplementary file 1 [file Table_1.DOCX]

**Supplementary Table S1. Database-Specific Search Strategies**

**Search date:** 31/03/2026
**Language restrictions:** None
**Date restrictions:** None

| **Database** | **Search Strategy** |
| --- | --- |
| **PubMed/MEDLINE** | ("digital eye strain" OR "computer vision syndrome" OR "visual display terminal syndrome" OR asthenopia OR "eye strain" OR "ocular strain" OR CVS) AND (adolescen* OR teen* OR youth OR "high school" OR "middle school" OR "school-aged") AND (prevent* OR interven* OR strateg* OR "20-20-20" OR "twenty-twenty-twenty" OR "blue light" OR blue-light OR ergonom* OR "blink train*" OR "screen break*" OR "screen time" OR "eye exercise*" OR "vision therapy" OR "anti-glare" OR "blue-blocking" OR filter* OR "artificial tears") |
| **Embase** | ('digital eye strain' OR 'computer vision syndrome' OR asthenopia OR 'eye strain' OR 'ocular strain') AND (adolescent* OR teenager* OR youth OR 'school aged') AND (prevention OR intervention OR strategy OR ergonomics OR 'blue light' OR 'screen break*' OR 'blink training' OR 'screen time' OR filters OR 'artificial tears') |
| **Scopus** | TITLE-ABS-KEY ("digital eye strain" OR "computer vision syndrome" OR asthenopia OR "eye strain") AND TITLE-ABS-KEY (adolescent* OR teen* OR youth OR "school-aged") AND TITLE-ABS-KEY (prevent* OR interven* OR ergonom* OR "blue light" OR "screen break*" OR "blink train*" OR filters OR "artificial tears") |
| **Web of Science** | TS=("digital eye strain" OR "computer vision syndrome" OR asthenopia OR "eye strain") AND TS=(adolescent* OR teen* OR youth OR "school-aged") AND TS=(prevent* OR interven* OR ergonom* OR "blue light" OR "screen break*" OR "blink train*" OR filters OR "artificial tears") |
| **CINAHL** | ("digital eye strain" OR "computer vision syndrome" OR asthenopia OR "eye strain") AND (adolescent* OR teen* OR youth OR school-aged) AND (prevention OR intervention OR ergonomics OR "blue light" OR "screen breaks" OR "blink training" OR filters OR "artificial tears") |
| **PsycINFO** | ("digital eye strain" OR "computer vision syndrome" OR asthenopia OR "eye strain") AND (adolescent* OR teen* OR youth) AND (prevention OR intervention OR behavior* OR ergonomics OR "screen break*" OR "screen time" OR "blink training") |
| **Google Scholar** | ("digital eye strain" OR "computer vision syndrome") adolescent intervention prevention |
| **ClinicalTrials.gov** | Condition/Disease: Digital Eye Strain OR Computer Vision Syndrome; Population: adolescents, youth, students; Intervention: prevention, ergonomics, filters, artificial tears, education |
| **WHO ICTRP** | Digital eye strain OR computer vision syndrome AND adolescent OR youth AND intervention OR prevention |

Searches combined controlled vocabulary terms (e.g., MeSH in MEDLINE and Emtree in Embase) with free-text keywords. Search syntax was adapted for the requirements of individual databases. Reference lists of included studies and relevant reviews were manually screened to identify additional eligible studies. Grey literature sources included Google Scholar (first 200 results), ProQuest Dissertations and Theses, OpenGrey, ClinicalTrials.gov, and the World Health Organization International Clinical Trials Registry Platform (ICTRP).
